# The passage of time in Iraq during the covid-19 pandemic

**DOI:** 10.1371/journal.pone.0266877

**Published:** 2022-04-14

**Authors:** Saad S. J. Alatrany, Ruth Ogden, Ashraf Muwafa Falaiyah, ‏Hasan Ali Sayyid ALdrraji, Abbas S. S. Alatrany

**Affiliations:** 1 Imam Ja’afar Al-Sadiq University, Baghdad, Iraq; 2 School of Psychology, Liverpool John Moores University, Liverpool, United Kingdom; 3 College of Education for Human Sciences Ibn Reshed, University of Baghdad, Baghdad, Iraq; 4 University of Information Technology and Communications, Baghdad, Iraq; 5 School of Computer Science and Mathematics, Liverpool John Moores University, Liverpool, United Kingdom; Unviersity of Sheffield, UNITED KINGDOM

## Abstract

The covid-19 global pandemic has influenced the day-to-day lives of people across the world. One consequence of this has been significant distortion to the subjective speed at which people feel like time is passing. To date, temporal distortions during covid-19 have mainly been studied in Europe. The current study therefore sought to explore experiences of the passage of time in Iraq. An online questionnaire was used to explore the passage of time during the day, week and the 11 months since the first period of covid-19 restrictions were imposed in Iraq. The questionnaire also measured affective and demographic factors, and task-load. The results showed that distortions to the passage of time were widespread in Iraq. Participants consistently reported a slowing of the passage of time for the day and the week during the pandemic in comparison to normal (i.e. before the pandemic). Participants also reported that it felt like longer than 11-months since the first lockdown began. The passage of time during the day and week were not predicted by any demographic, affective or task-load measures taken in the study. The perceived length of time since the first lockdown was however predicted by stress and change of life due to covid, with greater stress and greater change of life being associated with greater subjective lengthening of the pandemic. The findings indicate that whilst distortions to the passage of time during covid-19 appear to be a global phenomenon, the factors which predict temporal experience during the pandemic differ between countries and cultures.

## Introduction

The coronavirus-19 global pandemic has significantly impacted on most elements of day-to-day life for the majority of the global population. Government imposed restrictions attempting to slow the spread of the virus, often termed “lockdowns”, as well as individual choices to isolate from others, have impacted on the ways in which people work, shop, communicate and socialise. Unsurprisingly, such significant changes in social and economic activity have had significant impact on a range of psychological processes.

### The passage of time during the covid-19 pandemic

Objectively time passes at a constant linear rate, subjectively however time often feels like it is passing more quickly or slowly than normal leading to the sensation of time flying or dragging by [1, 2]. Such changes in the subjective speed at which time appears to pass are referred to as distortions to the passage of time.

Research conducted in Europe and South America suggests that a commonly reported consequence of life during covid-19 is distortion to the passage of time [3–12]. Studies conducted in the UK [3, 4], France [5, 6], Italy [7, 8], Uruguay [9] and Argentina [10], in which people were asked to compare speed at which time was passing during the pandemic to before the pandemic, have reported widespread and significant distortion to the passage of time during the covid-19 pandemic in comparison to prior to the covid-19 pandemic. Distortions are not limited to any particular age group or gender [3–8]. Nor are they unique to neurotypical groups, having also been observed in people with dementia with Lewy bodies [8]. Distortion to the passage of time during covid-19 therefore appears to be a global phenomenon experienced across populations.

Although distortions to the passage of time appear common during covid-19, there are notable differences in the way in which time distorted in the European states in which it has been studied. In France and Italy participants reported a slowing of the passage of time during covid-19 in comparison to normal (i.e. prior to covid-19) [5–8]. In the UK however, although less than 20% of participants reported time passing as normal during covid-19, participants were equally likely to report time passing more quickly than normal as more slowly than normal [3, 4] when asked about their experience of time during the day and the week. Covid-19 is not therefore associated with a universal slowing of the passage of time across the day and week. However, when people in the UK were asked about the 8-months since covid-19 restrictions were in place, participants overwhelmingly reported that the 8-month period felt longer than its actual duration, suggesting some evidence of universal slowing in the UK over longer periods [4].

Despite differences in the way in which time distorted across countries during the pandemic, there were some commonalities in the factors which influenced temporal experience in the different countries. In particular, in the UK, France and Italy, emotions associated with a lack of socialisation i.e. increased sadness [4, 5], boredom [3–7] and dissatisfaction with social interaction [3, 4] were associated with a slowing of the passage of time. There were also cross-country similarities in the factors which did not predict distortions to the passage of time, with studies conducted during the first few months of the pandemic showing no association between depression and anxiety and temporal experience in any country [3–12].

Differences in temporal experience across different countries and cultures, despite similarities in the factors which predict temporal experience, suggest that cross-cultural differences in the passage of time during covid-19 may arise from cultural and economic factors. These may be general i.e. differences in economic security, family structure and social norms, or they may be specific to temporal experience, for example, whether the culture is considered to be polychronistic or monochronistic [13, 14]. Polychronistic cultures, for example, are characterised as experiencing time flexibly and place less value on schedules. Conversely, monochronistic cultures are characterised as experiencing time as tangible and linear. Alternatively, they may emerge from cross-cultural differences in the effect of covid-19 on specific societal and economic structures and practices within a given country or culture.

To date, our understanding of the effect of covid-19 on temporal experience is largely limited to temporal experience in Europe and South America and during periods of lockdown [3–12]. It is therefore unclear whether the distortions observed in existing studies would also be observed in countries with significantly different cultural and economic profiles. To fully understand the impact of the covid-19 on temporal experience, it is therefore important to study the passage of time outside of these countries and cultures. The current study therefore sought to explore experiences of the passage of time during the covid-19 pandemic in the Republic of Iraq.

### Covid-19 in Iraq

The first Covid-19 case confirmed in Iraq was in Najaf, 180 KM to the south of Baghdad, on 24 Feb 2020 [15]. Consequently, the Iraqi government established The Higher Committee for Health and National Safety to be responsible of managing all issues related to control the spread of the Covid-19. On 17th of March 2020, the committee announced total lockdown measures which remained in place until 21^st^ April 2020. These included closing all public and private organizations, universities, schools, restricting transportation and restricting movement between cities [16]. From April 2020 until September 2020 a partial lockdown was imposed in which, between 8pm and 5am, travel was prohibited and shops and businesses were closed. During this period of partial lockdown two further periods of full lockdown were imposed during Eid al-Fitr and Eid al-Adha for 15 and 10 days respectively. From September 2020 to February 2021 lockdown was completely lifted in Iraq however partial lockdown was then reimposed during February 2021. Despite these restrictions, the total number of confirmed covid-19 cases in Iraq from 24 Feb 2020 until 28 May 2021 was 1,190,351 and there were 16,311 deaths [17].

In Iraq, as in many other countries, covid-19 and the associated restrictions on society have negatively impacted on health, wealth and wellbeing for many. Iraq’s economy contracted by approximately 10.4% in 2020 [18]. Although this was largely due to reductions in demand for oil, it is also thought to be due to covid-19 itself [18]. This has impacted negatively on many small and medium sized enterprises [19] and led to a significant increase in rates of poverty [20]. Despite this however, unemployment levels have remained comparable to pre-pandemic levels [21]. In addition to these economic consequences, covid-19 has also been associated with increased levels of depression and anxiety in Iraq’s general population and in particular in women [22]. Furthermore, violence against women has increased on pre-pandemic levels [23]. However, despite such significant and prolonged changes in day-to-day there has been little assessment of how these changes have impacted on broader cognition.

### The current study

The current study aimed to establish whether the covid-19 pandemic had distorted the passage of time for people in Iraq. To assess the effect of covid-19 on the passage of time in Iraq, a modified version of the passage of time questionnaire developed in Ogden (2020, 2021) [3, 4] was translated into Arabic by the research team. The questionnaire required participants to report their experience of the passage of time during the day of study (POTJ-day) and the week of study (POTJ-week). Participants also indicated the subjective length of the 11 months since lockdown in Iraq began (POTJ-11 months) i.e. longer than 11 months or shorter than 11 months. Depression, anxiety and stress were measured using the Arabic version of the DASS-21 [24] and task load was assessed using the NASA-TLX [25]. Other measures in the questionnaire explored demographics, levels of physical activity, satisfaction with social interaction, compliance with restrictions, extent to which life changed during the pandemic and perceived risk from covid. Participants completed the questionnaire between 20^th^ April 2021 and 30^th^ April 2021 during which time Iraq was in partial lockdown meaning that between 8pm and 5am a curfew was in place prohibiting travel and closing shops and businesses.

Given the wide-ranging impact of covid-19 on day-to-day life in Iraq, it is plausible that Iraqi’s may have experienced distortions to the passage of time comparable to those observed in other studies [3–12]. However, cultural differences in the way in which time is conceptualised between Middle Eastern and Northern European countries may have influenced the way in which covid-19 has impacted temporal experience in Iraq. Hall (1973, 1989) [13, 14] describes temporal experience as either monochronic or polychronic. Monochronic cultures typically experience time as tangible and linear. As a consequence, events are scheduled and conducted in linear manner, often one at a time. Polychronic cultures typically experience time flexibly and as a consequence, schedules are thought have less value than in polychronic cultures. Monochronic culture is typically associated with Northern Europe and North America whereas polychronic culture is associated with Latin America and the Middle East. Iraq’s association with polychronic temporal experience may therefore mitigate the effect of covid-19 on the passage of time. For example, increased temporal flexibility associated with polychronic temporal experience may provide resilience against the effects of covid-19 and its associated restrictions on distortions to the passage of time.

Based on studies conducted in Europe and South America [3–12], it was expected that distortions to the passage of time would be widespread in Iraq. However, because Iraq is thought to have a polychronic temporal culture [13, 14] and because only a partial lockdown was in force during the period of study, the severity of distortions to the passage of time and the factors which predict temporal experience in Iraq were expected to differ from those observed in Europe.

## Method

### Participants

Five hundred sixteen participants were recruited through volunteer sampling. Participants were recruited using paid posts on social media networks. The posts were configured to recruited participants from the different cities in Iraq and people of different genders and ages. Participants completed the questionnaire between 20^th^ April 2021 and 30^th^ April 2021. Six participants were excluded from the study because the failed to answer one or more questions. The final sample was therefore 510 participants (45% male, 55% female). The age of participants ranged from 18 to 70 years (*M* = 34.64, *SD* = 12.85). Table 1 shows demographic information for the sample. The study was approved by Liverpool John Moores University Research Ethics Committee and all participants gave informed written consent. The study was conducted in accordance with the principles expressed in the Declaration of Helsinki.

**Table 1:** Descriptive statistics of the proportion of participants in different demographic groups and the mean POTJ for each group.

	Mean (SD) % [N]	Mean POTJ-day (SD)	Mean POTJ–week (SD)	Mean POTJ-11 months (SD)
*Age (years)*	34.64 (12.85)			
*Young < 26*	31.50 [179]	3.77 (1.43)	3.64 (1.38)	4.08 (1.18)
*Middle aged*	59.40 [303]	3.90 (1.41)	3.99 (1.44)	3.66 (1.24)
*Older >60*	5.50 [28]	3.25 (0.80)	3.50 (1.07)	3.79 (1.34)
*Gender*				
*Male*	55.50 [283]	3.76 (1.42)	3.77 (1.34)	3.79 (1.21)
*Female*	45.50 [227]	3.87 (1.36)	3.89 (1.43)	3.83 (1.26)
*In an at risk group*				
*Yes*	24.70 [126]	3.98 (1.51)	3.92 (1.44)	3.90 (1.29)
*No*	23.70 [121]	3.79 (1.31)	3.81 (1.36)	3.75 (1.28)
*Unsure*	51.60 [263]	3.75 (1.37)	3.81 (1.39)	3.80 (1.91)
*Known deceased*				
*Yes*	55.68 [284]	3.83 (1.41)	3.88 (1.41)	3.89 (1.25)
*No*	44.32 [226]	3.18 (1.37)	3.80 (1.38)	3.75 (1.22)
*Employment status*				
*Employed full time in office*	34.90 [178]	3.74 (1.34)	3.78 (1.35)	3.67 (1.23)
*Employed full time at home*	12.50 [64]	3.97 (1.38)	4.27 (1.55)	3.72 (1.29)
*Employed part time*	13.30 [68]	3.81 (1.28)	3.85 (1.26)	3.72 (1.22)
*Unemployed looking for work*	2.50 [13]	4.15 (1.82)	3.62 (0.87)	4.08 (1.12)
*Unemployed not looking for work*	1.70 [8]	3.00 (0.00)	2.50 (0.71)	5.00 (0.00)
*Student*	35.10 [179]	3.84 (1.45)	3.77 (1.43)	4.00 (1.22)
*Depression*	7.52 (5.02)			
*Normal*	30.60 [156]	3.62 (1.17)	3.69 (1.25)	3.78 (1.14)
*Moderate*	43.30 [221]	3.94 (1.35)	3.90 (1.32)	3.81 (1.20)
*Severe*	26.10 [133]	3.86 (1.64)	3.90 (1.65)	3.86 (1.40)
*Anxiety*	5.42 (4.73)			
*Normal*	43.30 [221]	3.71 (1.23)	3.70 (1.24)	3.80 (1.15)
*Moderate*	26.90 [137]	3.82 (1.29)	3.93 (1.46)	3.81 (1.25)
*Severe*	29.80 [152]	3.97 (1.59)	3.96 (1.51)	3.84 (1.35)
*Stress*	8.28 (5.27)			
*Normal*	49.00 [250]	3.79 (1.27)	3.82 (1.31)	3.72 (1.17)
*Moderate*	29.60 [151]	3.91 (1.38)	3.93 (1.37)	3.91 (1.18)
*Severe*	21.40 [109]	3.82 (1.64)	3.73 (1.60)	3.91 (1.44)
*Number of cohabitants*	5.68 (2.81)			
*Task-load*	17.25 (3.89)			
*Social satisfaction*	2.59 (1.21)			
*Physical activity*	2.42 (1.09)			
*Changed routine*	3.67 (1.24)			

### Measures

The questionnaire was delivered online via Google forms. The questionnaire was a modified version of the passage of time questionnaire developed in Ogden (2020) [3, 4]. The questionnaire explored demographics, experiences of covid-19, levels of physical activity, social satisfaction and compliance with restrictions. Mood was assessed using the DASS-21 [24] and task load was assessed using the NASA-TLX [25]. All questions were translated in Arabic. The questionnaire took approximately 5 minutes to complete.

#### Demographic questions

Participants stated their age, gender, employment status, whether they were in a high-risk category for Covid-19 and how many people they lived with.

#### Passage of time judgements

The following questions were posed about the passage of time.

1 *“Thinking about today*, *how quickly has time felt like it is passing in comparison with normal (i*.*e*. *before lockdown)*?*”*
2 *Thinking about this week*, *how quickly has time felt like it was passing in comparison to normal (i*.*e*. *before lockdown)*?

Participants responded using the following 7 point Likert scale: 1. Extremely slow, 2. somewhat slower, 3. a little slower, 4. as normal, 5. a little faster, 6. somewhat faster, 7 extremely fast. A higher score therefore indicated a faster passage of time.

A further question explored the subjective length of time since the first covid-19 lockdown commenced in Iraq.

3 *It is 11 months since Iraq first went into lockdown*. *It feels like*

Participants responded using a 5 point likert scale: 1. a lot shorter, 2. somewhat shorter, 3. about 11 months, 4. somewhat longer, 5. a lot longer.

#### Lifestyle questions

To measure social satisfaction participants were asked to rate how “Since the Covid-19 lockdown, how satisfied are you with your daily level of social interaction?” using a 5 point Likert scale in which a high score indicated greater satisfaction. To measure physical activity, participants rated “Since the Covid-19 lockdown, how would you describe your level of physical activity? Using a 5 point Likert scale in which a high score indicated greater activity. Finally, participants also used a 5 point Likert scale to rate to what extent they agreed that: "My daily routine has changed a lot as a result of the Covid-19 lockdown? Here, a high score indicated greater agreement.

#### DASS-21

The Arabic version of the DASS-21 [24] was used to measure depression, anxiety and stress. The 21-item questionnaire has three seven item subscales which measure depression, anxiety and stress. Responses are provided by indicating the extent to which each item reflected the participants experience: (1) did not apply to me at all; (2) applied to me to some degree; (3) applied to me to a considerable degree; and (4) applied to me very much. Although the DASS-21 is not a diagnostic tool, scores from the DASS-21 can be doubled to enable classification as normal, moderate or severe using the following cut-offs: depression; normal < 9, moderate 10–20 and severe > 21, anxiety; normal < 6, moderate 9–14 and severe > 15 and stress; normal < 10, moderate 11–26 and severe > 27. Cronbach’s alpha for the 21 item DASS questionnaire was 0.94, 0.85 for the depression subscale, 0.84 for the anxiety subscale and 0.87 for the stress subscale.

### National Aeronautics and Space Administration-Task Load Index (NASA-TLX)

The NASA-TLX [25] is a measure of subjective workload. It uses six single item questions to measure: mental demands, physical demands, temporal demands, personal performance, effort and frustration. In the current study, a modified version of the NASA-TLX was used to assess the subjective workload of an average day. Participants were asked to rate each of the six items, in terms of their average day during the lock-down period, using a 5-point Likert scale in which a high score indicated greater task demands. Cronbach’s alpha for NASA-TXL questionnaire was 0.70.

### Data analysis

The analysis strategy replicated that used in Ogden (2020; 2021) [3, 4]. Non-parametric tests were used because the passage of time judgments were ordinal data. Kruksal-Wallis tests and Mann Whitney U tests were used to establish the effect of age, gender, personal risk, employment and knowing someone who had died from covid-19 on POTJs. In this analysis, as in Ogden (2020) [3, 4], age was classified into three groups; young adults (25 years and under), middle aged adults (26–59 years) and older adults (aged 60 years and over). Unlike in Ogden (2020; 2021) [3, 4], cohabitation status was not analysed because very few people reported living alone. To assess the relationship between the passage of time and measures of affect (DASS-21 depression, anxiety and stress scores, satisfaction with social interaction), task load (NASA-TXL scores and rating of physical activity), conformity and age, Spearman’s correlations were conducted. Unlike in Ogden (2020; 2021) [3, 4], due to the low number of people living alone and high variability of the number of people cohabiting, number of cohabitants was also correlated against POTJs. Finally, to assess whether these factors were predictive of POTJ’s, separate ordinal logistical regression analyses were conducted for POTJ-day and POTJ-week and POTJ-11 months.

## Results

### The passage of time

**Fig 1:**
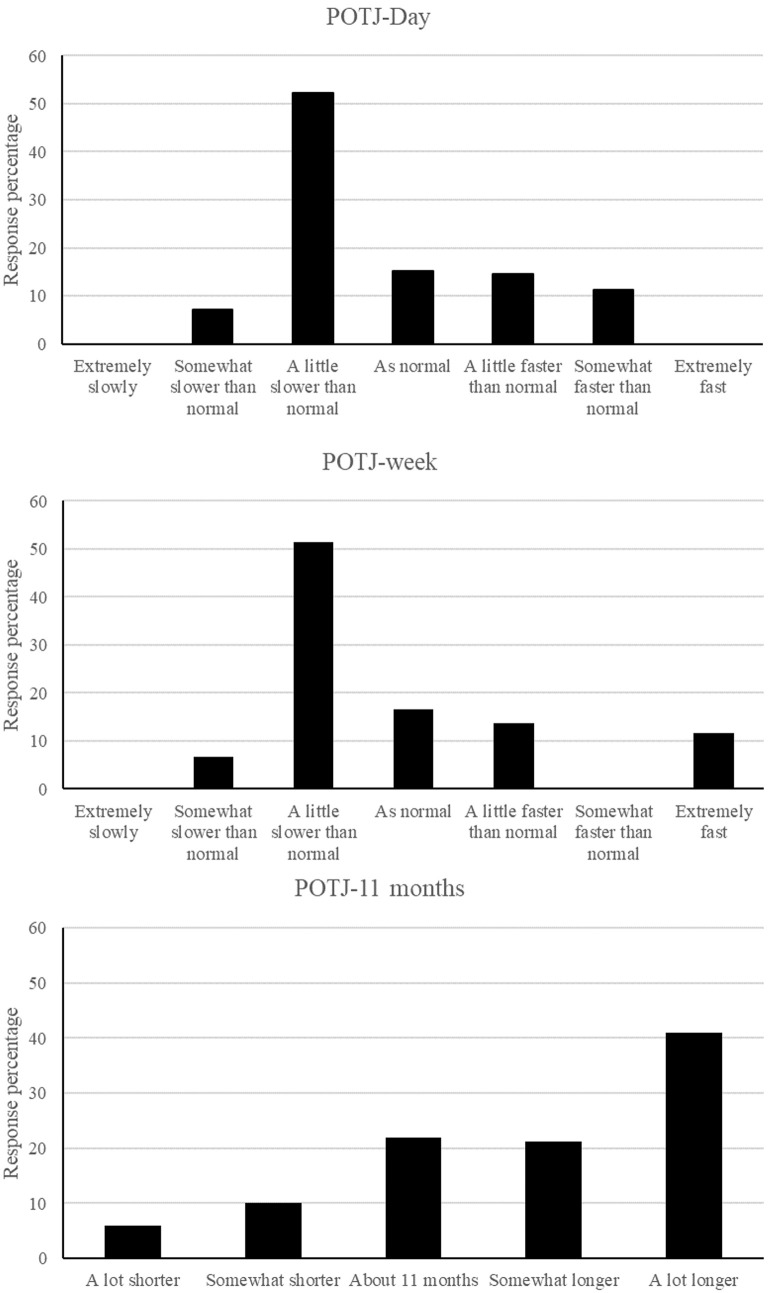
The frequency of responses for each Likert point for the day judgement (upper panel), week judgment (middle panel) and 11-month judgment (lower panel).

Fig 1 shows the distribution of responses for the day (upper), week (middle) and 11 months (lower) passage of time judgments. Examination of Fig 1 suggests that distortion to time was prevalent in Iraq during 11 months since lockdown commenced. For POTJ-day and POTJ-week, fewer than 20% of participant reported that time passed as normal during lockdown. Instead, participants overwhelming reported a slowing of the passage of time during the day and week during lockdown in comparison with before. For POTJ-11 month, 22% of participants reported that the restrictions felt like they had been in place for about 11 months, 16% reported that it felt like less time than that and 62% reported that it felt longer than 11 months.

### The effect of demographic factors on POTJ

Table 1 shows mean passage of time judgments expressed as a function of age group, gender, personal risk, occupation and whether someone close to them had died. Table 2 shows analysis of this data using Kruksal-Wallis tests and Mann Whitney U tests. Examination of Table 2 suggests that there was a significant effect age group for POTJ-week and POTJ-11 month. POTJ-week was faster for middle aged than younger people (*p* < .05). POTJ-11 was shorter for the younger people than those middle aged (*p* < .05). There was no significant effect of gender, perceived personal risk of covid, knowing someone who had died or employment status on any measure of POTJ.

**Table 2:** Outcomes of the analysis of the effect of demographic factors on POTJ’s.

	POTJ-Day		POTJ-Week		POTJ- 11 months	
*Variable*	Statistic	*p*	Statistic	*p*	Statistic	*p*
*Age group*	5.79	.06	9.72	.01	16.03	.001
*Gender*	29987.00	.16	30825.00	.40	57118.00	.58
*Perceived risk*	5.29	.07	4.41	.11	.81	.67
*Employment*	2.84	.59	8.20	.08	9.49	.06
*Known deceased*	31628.50	.76	30955.50	.46	29601.50	.11

### Correlates and predictors of POTJ

Table 3 shows correlation coefficients for the relationships between POTJs and measures of affect, task load, social satisfaction, physical activity, change in daily routine, compliance with restrictions and the number of cohabitants. There were no significant correlations between POTJ-day and POTJ-week and any of the measures of affect, task load, social satisfaction, physical activity, change in daily routine, compliance with restrictions and the number of cohabitants. For POTJ-11 months, significant negative correlations were observed with age, social satisfaction and physical activity and significant positive relationships were observed with change to daily life, number of cohabitants and stress. A longer lockdown was therefore associated with younger age, less social satisfaction and less physical activity, more change to life, greater numbers of cohabitants and greater stress.

**Table 3:** Correlation coefficients between POTJs, age, measures of affect, load, compliance, number of cohabitants and change to life.

	POTJ- Day	POTJ-Week	POTJ-11 months
***Age***	-.02	.05	-.14**
***Social satisfaction***	.01	.10	-.09*
***Change to daily life***	-.01	.03	.12**
***Number of cohabitants***	-.05	-.03	.09*
***Level of physical Activity***	.06	.01	-.13**
***Depression***	.002	.01	.07
***Anxiety***	.04	.05	.03
***Stress***	-.01	-.03	.11*
***Task-load***	.01	-.03	-.06
***Compliance***	-.03	-.06	-.02

* = *p* < .05,

** = *p* < .01.

To establish the effect of demographic factors, measures of affect, task-load and the other measured variables on POTJ-day, POTJ-week and POTJ-11, ordinal regression with proportional odds was conducted separately for each POTJ. Table 4 shows the odds ratios for each variable with 95% confidence intervals.

**Table 4:** Wald, odds ratios and 95% confidence intervals from the ordinal regressions for POTJ-day, POTJ-week and POTJ-11 months.

		*POTJ-day*			*POTJ-week*			*POTJ- 11 month*		
		*Wald*	*Odds Ratio*	*95% CI*	*Wald*	*Odds Ratio*	*95% CI*	*Wald*	*Odds Ratio*	*95% CI*
*Age*		.73	.99	.97–1.01	.005	.10	.98–1.02	.28	1.10	.98–1.01
*Gender*	Female (reference)									
	Male	2.26	.75	.52–1.09	3.01	.72	.40–1.04	.07	1.05	.73–1.50
*Known deceased*	Yes (reference)									
	No	.06	.96	.68–1.40	.09	.95	.67–1.34	2.12	.78	.55–1.09
*Perceived greater risk*	Unsure (reference)									
	No	.16	1.09	.71–1.67	.01	1.02	.67–1.56	.06	1.05	.70–1.59
	Yes	2.58	1.42	.93–2.16	.24	1.11	73–1.70	2.35	1.39	.91–2.11
*Employment status*	Employed full time home (reference)									
	Employed full time in office	2.09	.67	.39–1.15	5.56	.52	.31-.90	.33	.86	.51–1.45
	Employed part time	.76	.75	.39–143	2.40	.60	.32–1.14	.01	.97	.52–1.81
	Unemployed looking for work	.01	1.00	.32–3.10	.81	.59	.19–1.86	1.28	1.95	61–6.20
	Student	2.12	.62	.33–1.18	6.71	.43	.23-.81	1.15	1.41	.75–2.64
*Socialisation satisfaction*		.20	.97	.83–1.12	.01	1.01	.87–1.17	.49	.95	.82–1.10
*Number of cohabitants*		.99	.97	.91–1.03	.02	100	.94–1.06	.68	1.03	.97–1.09
*Depression*		.02	1.00	.94–1.06	.11	1.01	.95–1.08	1.63	.96	.90–1.02
*Anxiety*		1.78	1.04	.98–1.10	4.24	1.06	1.00–1.13	1.71	.96	.91–1.02
*Stress*		1.13	.97	.91–1.03	4.94	.93	.88-.99	5.18*	1.07	1.01–1.14
*Task-load*		.02	1.00	.96–1.05	.11	.99	.94–1.04	.01	1.00	.95–1.05
*Physical activity*		2.55	1.17	.97–1.41	.49	1.07	.89–1.30	1.76	.88	.73–1.06
*Change of routine*		.26	1.04	.90–1.19	1.24	1.08	.94–1.25	7.53**	1.21	1.06–1.39
*Compliance*		.76	.92	.77–1.11	4.31	.82	.69-.99	.27	.95	.80–1.14

* = *p* < .05,

** = *p* < .001.

No significant model fits were observed for POTJ-day χ^2^(18) = 16.06, *p* = .059 or POTJ-week χ^2^(18) = 22.75, *p* = .20. For POTJ-11 month the model was a statistically significant, χ^2^(18) = 34.60, *p* = .01 fit for the data, with pseudo R squared values of .02–.07. Only stress and change to daily life were significant predictors of POTJ-11months. The 11 months of restrictions feeling long was therefore associated with greater change of life and greater stress.

Together these findings suggest that although time was distorted for POTJ-day, POTJ-week and POTJ-11 months, the factors measured in this study could only explain a significant proportion of the variance in the longer judgment (POTJ-11 months), thus implying that different factors influenced shorter and long term passage of time in this population.

## Discussion

This study aimed to explore the experience of the passage of time for people in Iraq during the covid-19 pandemic. In addition, the study aimed to identify factors which contributed to distortions to the passage of time during the pandemic in Iraq.

The results show that distortions to the passage of time were commonplace for people in Iraq during the pandemic. Fewer than 20% of participants reported that the day and the week passed at a speed comparable to before the pandemic. Instead, participants overwhelmingly reported a slight slowing of the passage of time for the day and the week during the pandemic in comparison with before. Distortions to the perceived length of the pandemic were also widespread, with over 62% of respondents indicating that it felt like longer than 11 months since the beginning of the first lockdown in Iraq. These findings therefore support the hypothesis that distortions to the passage of time would be widespread in Iraq during the covid-19 pandemic.

Although there was significant distortion to the passage of time during the day and the week for people in Iraq during the pandemic, the measures of mood, demographics, task-load, social satisfaction, physical activity and compliance taken in this study were not predictive of short-term (day and week) temporal experience in Iraq during the pandemic. The measures taken were however predictive of longer-term temporal experience in Iraq’s pandemic with the perceived length of the 11-months since the pandemic began being predicted by the extent to which life had changed due to the pandemic and levels of stress. Specifically, the 11-months since restrictions began felt longer for those whose lives had changed to a greater extent as a result of the pandemic and for those who were experiencing greater amounts of stress.

The association between greater life change and a longer perceived pandemic may be explained by the role of memory in temporal judgements. It has been suggested is that we use the number of memory representations or the amount of contextual change within an event to retrospectively determine its duration [26–28]. This suggestion is supported by previous research which has demonstrated that greater memory load and greater contextual change are associated with longer retrospective estimates of an events length [26–28]. In the context of the pandemic, greater change to life because of the pandemic is likely to be associated with the formation of a greater number of memory representations and potentially with greater contextual change. Memory formation is therefore a plausible mechanism through which change to life may have influenced the perceived length of the pandemic.

Interestingly, although stress levels were predictive of the perceived length of the pandemic, measures of depression and anxiety were not predictive of the passage of time for the day, week or 11-month period. This is unlikely to be because levels of anxiety and depression were too low to have an effect on perceived duration as a high proportion of participants reported moderate (43.30%) or severe (26.10%) levels of depression and moderate (26.90%) or severe (29.80%) levels of anxiety. Indeed, the proportion of participants reporting severe depression and stress in this study is far greater than that reported in the UK during the pandemic [3, 4]. Although Ogden (2021) [4] found depression to be predictive of temporal experience later into the UK lockdown, the majority of existing studies have failed to observe an effect of anxiety and depression on the passage of time during the pandemic [3, 5–8]. This study therefore adds to a growing body of evidence that depression and anxiety do not consistently distort the passage of time. The absence of consistent effects of anxiety and depression on the passage of time during the global pandemic is surprising. Previous real-world studies of the passage of time have indicated that affect distorts the passage of time, with positive affect being associated with a quicker passage of time and negative affect being associated with a slower passage of time [29, 30]. Furthermore, clinical studies indicated that a slowing of the passage of time as a consistent feature of depressive episodes for people with depression [31–34]. One possible explanation for this inconsistency is that the influence of depression and anxiety on temporal experience is somewhat attenuated when so many other significant changes are occurring in life (i.e. other changes due to the pandemic). This suggestion is supported by observations that “change to life” because of the pandemic is a predictor of distortions to the passage of time in studies conducted later into the pandemic [4].

Comparison of these findings from Iraq with those from studies conducted in the UK, Italy, France, Brazil and Uruguay confirm that distortions to the passage of time during the day and the week during covid-19 are not limited to these territories [3–12]. Instead, the findings suggest that distortions to the passage of time are a global feature of the covid-19 pandemic. However, comparison of the nature of the distortions to the passage of time in Iraq, the UK, Italy, France, Brazil and Uruguay [3–12] indicate that although distortions to time are ubiquitous there are cross-cultural differences in their manifestation. Specifically, in Iraq, France and Italy [5–8], covid-19 was associated with a predominant slowing of the passage of time during the day and week. Similarly, in Brazil, people experienced an expansion of time [10]. In the UK [3, 4] however, slowing and speeding up of the passage of time during the day and week were equally likely. Furthermore, there also appear to be significant cross-cultural differences in the factors which predict POTJ-day and POTJ-week. In the UK, France and Italy temporal experience during the day and week was predicted by affective factors such as sadness, social dissatisfaction and stress and task-load factors such as boredom. In Iraq however, these factors were not associated with changes in the passage of time during the day and the week.

Whilst it is unclear why POTJ-day and week in Iraq were unrelated to affect and task-load, we speculate that this may reflect the timings during which the studies were conducted. Studies conducted in the UK, France and Italy were conducted during periods of full lockdown in which there were significant restrictions curtailing the activities of residents. These typically included the total closure of schools and non-essential shops, travel restrictions and the prohibition of social mixing between households. At the point of data collection in Iraq however only a partial lockdown was in force in which there was a night-time curfew and travel restrictions between cities. The absence of significant restrictions on daytime life in Iraq may therefore have limited the effect of social satisfaction and task-load on temporal experience because these factors were broadly unaffected by covid-19 at that particular point in time. This therefore raises the possibility that different factors may have predicted temporal experience in Iraq if more stringent restrictions were in place. A further possibility is that a more polychronic culture in Iraq [13, 14], in which time is experienced more flexibly, meant that factors other than those measured in this study were determinant of temporal experience. A final speculative possibility is that the recent history of instability in Iraq, notably the US-UK invasions, made the population more resistant to the effects of significant change in daily life and emotional distress on broader perception and cognition. This may therefore have limited the capacity for these factors to influence temporality. Future research should therefore seek to measure a broader range of predictive factors to understand the drivers of temporal experience in Iraq and other cultures associated with a polychronic temporal culture.

The observation of distortions to the passage of time in Iraq during a period in which a full lockdown was not in place does however suggest that distortions to the passage of time are not dependent on “full lockdowns” in which most forms of socialisation are prohibited. Instead, they can occur in situations in which social interaction is preserved but other elements of life are restricted. This raises the possibility that as societies adapt to learn to live with covid-19, distortions to the passage of time may continue for even in the presence of more minor restrictions.

A further possibility is that changes in time experience during and after the pandemic may be a critical factor in individual and societal recovery from the pandemic. Temporal vertigo [35] describes the feelings of confusion and anxiety about where we are in the timeline of past, present and future after a crisis or disaster. Feelings of temporal vertigo are consequential, typically being associated with poorer outcomes and recovery [35]. One speculative possibility is that distortions to the passage of time may contribute towards the experience of temporal vertigo because greater distortion to the passage of time over short (days and weeks) and long (months) epochs may exacerbate to the sensation of being lost in time. It is therefore important that research seeks to understand how temporal experience contributes to wellbeing and recovery.

### Limitations

Whilst this study demonstrates that widespread distortion to the passage of time has occurred in Iraq during the covid-19 pandemic, it has been broadly unable to identify the psychological and demographic factors which determined temporal experience. This is despite using measures which have predicted the passage of time during the pandemic in other countries [3, 4]. Whilst this implies that cross-cultural differences exist in the factors which determine temporal experience, it also highlights the importance of identifying culturally relevant measures in order to determine temporal experience in different countries and cultures. Future research should therefore explore how a broader range of culturally relevant factors determine how and why time distorts. This may include measures of factors known to significantly affect life at a national level e.g. political and economic stability, and autonomy in behaviour and decision making, as well as temporally specific measures such as the extent to which an individual is poly or monochronic. However, to fully understand the culturally specific factors which affect time experience, in-depth ethnographic and qualitative research may be required to explore the relationships between culture, behaviour and temporality in traditionally under researched groups.

Our ability to make cross-cultural comparisons between the findings of this study and those observed in other countries is also limited by the differing restrictions in place at the time of data collection in the different studies. Specifically, whilst other studies have been conducted during “full” lockdowns, only a partial lockdown was in place during the current study. These differences are likely to have contributed to differences in the manifestation and predictors of temporal distortions across studies. Future research should therefore seek collect data during periods of similar restriction.

### Conclusion

The results of this study suggests that people in Iraq experienced a consistent slowing of the passage of time during the covid-19 pandemic. They also experienced the 11 months since the pandemic began as feeling like longer than its actual duration. Although the current study failed to identify the factors which predicted temporal distortions during the day and the week, a lengthening of the perceived duration of the pandemic was predicted by increased stress and greater change to life. These findings confirm that distortions to the passage of time are not unique to European and South American cultures but instead appear to be a global phenomenon. Furthermore, the presence of distortions to the passage of time during a partial lockdown demonstrate that total lockdown is not required for time to distort. The presence of significant distortions to time 11 months into the covid-19 pandemic demonstrates the continued impact of the pandemic on broader forms of cognitive function and the need for continued research to understand the long-term implications of the pandemic on cognition.

## Supporting information

S1 Data (SAV)
